# Supplementary material for: Host adaptation in gut Firmicutes is associated with sporulation loss and altered transmission cycle
Source: Genome Biol. 2021 Aug 5;22:204. doi: 10.1186/s13059-021-02428-6 (PMC8340488; doi:10.1186/s13059-021-02428-6)
Supplement: Supplementary file 1 — Additional file 1: Figure S1. Environmental distribution of genomes from Firmicutes bacteria. Figure S2. Prediction of sporulation capability in Firmicutes. Figure S3. Phenotypic validation of sporulation capability predictions. Figure S4. Genome reduction and metabolic specialization during host-adaptation by gut Firmicutes. Figure S5. Erysipelotrichaceae Former-Spore-Formers have a reduced carbohydrate metabolism profile compared to Erysipelotrichaceae Spore-Formers. Figure S6. Former-Spore-Formers are less prevalent than Spore-Formers in gut metagenomes from the same country. Figure S7. Spore-formers contribute more to beta-diversity compared to non-spore-forming bacteria in the human intestinal microbiota. [file 13059_2021_2428_MOESM1_ESM.docx]

**Additional File 1, Contains Figures S1 to S7:**

**
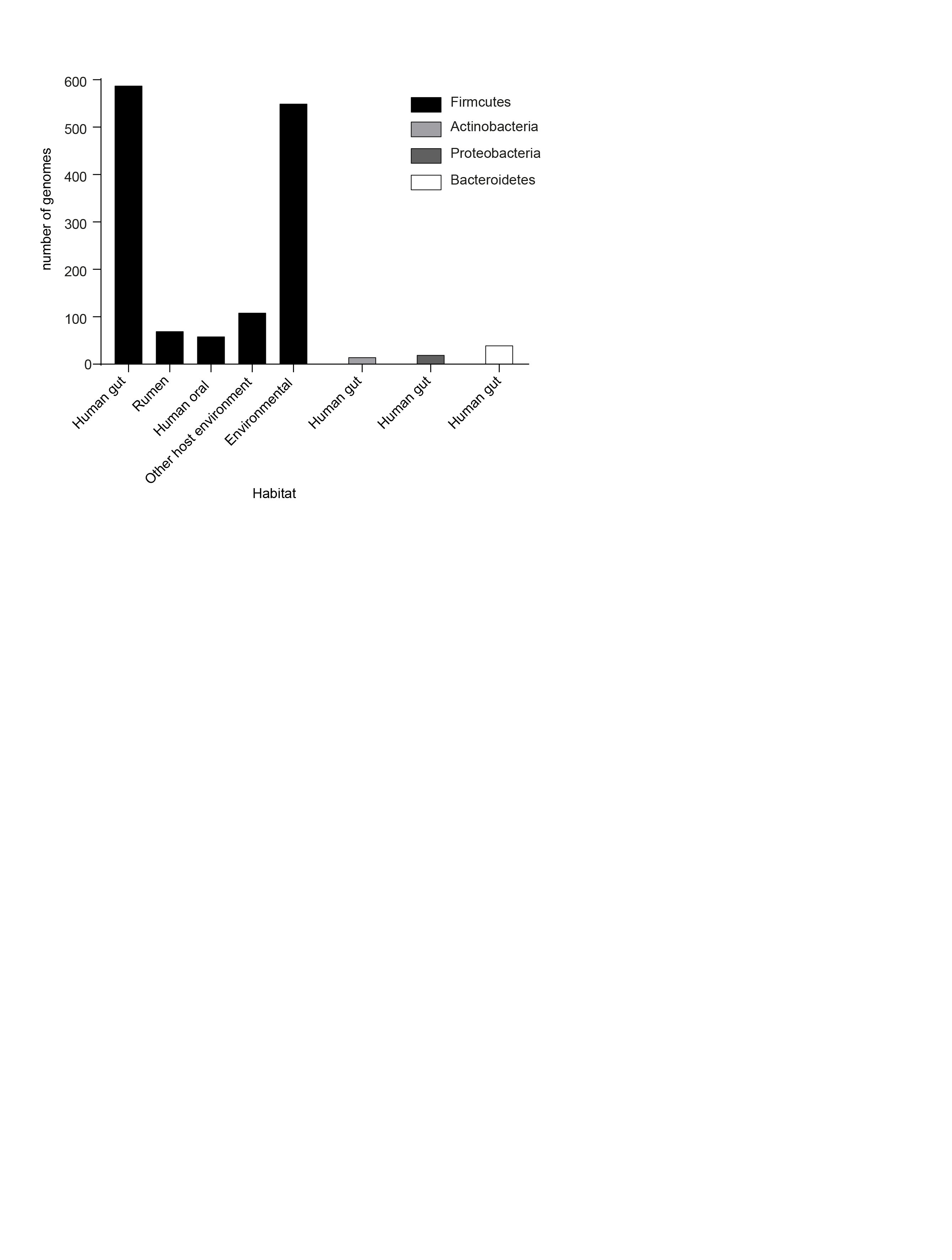
**

**Figure S1: Environmental distribution of genomes from Firmicutes bacteria**

Firmicutes genomes (n=1358) are derived from bacteria present in a diverse range of host and non-host-associated habitats**.** Actinobacteria (n=14), Proteobacteria (n=19) and Bacteroidetes (n=39) genomes, all derived from gut bacteria, were included in analysis for comparative purposes.


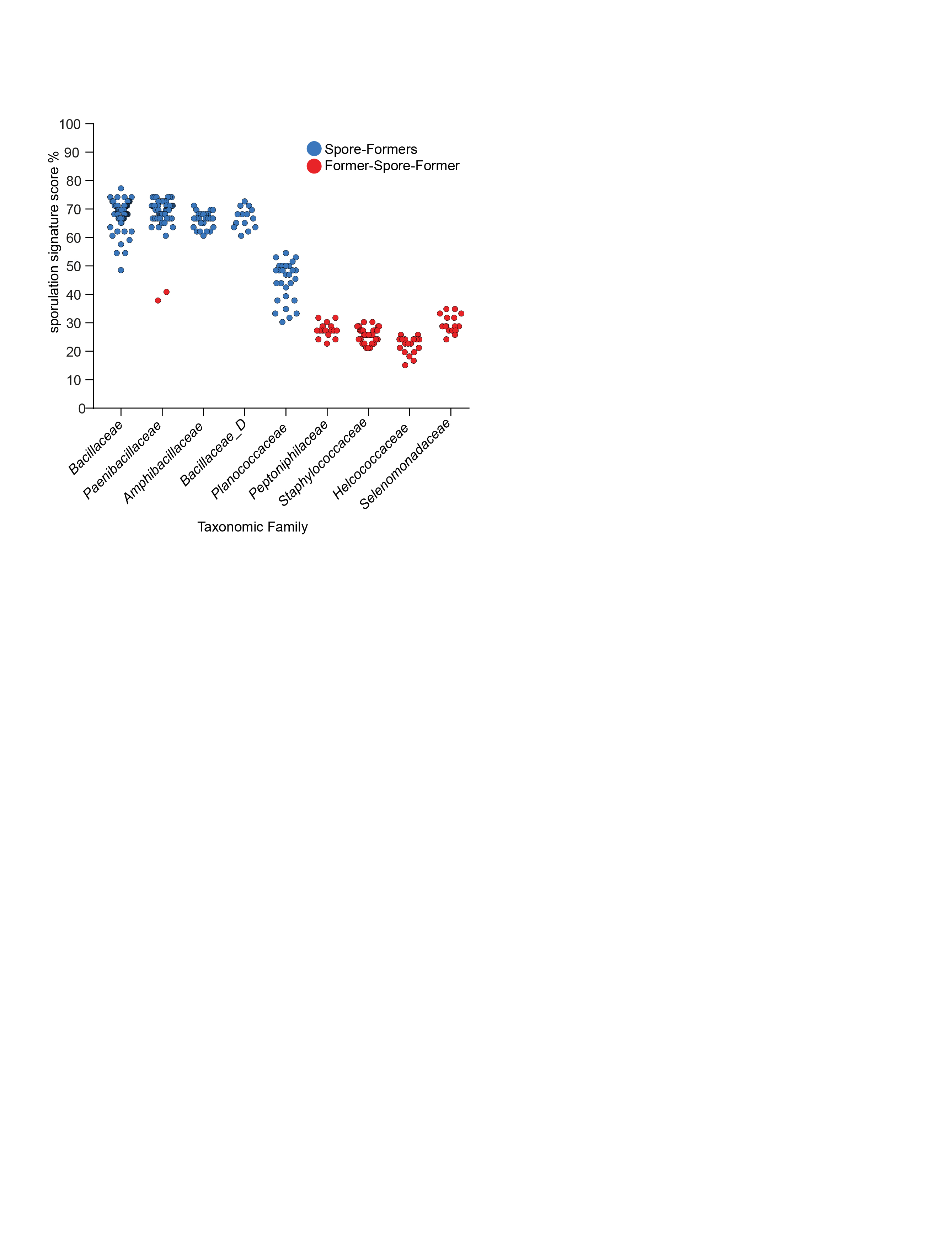


**Figure S2: Prediction of sporulation capability in Firmicutes**

**(a)** Prediction of sporulation capability in Firmicutes families based on the presence of 66 sporulation associated genes. These families have either a high sporulation signature score (blue dots) (classified as Spore-Formers), a low sporulation signature score (red dots) (classified as Former-Spore-Formers) or a bimodal pattern with both high and low scoring sporulation signature genomes.


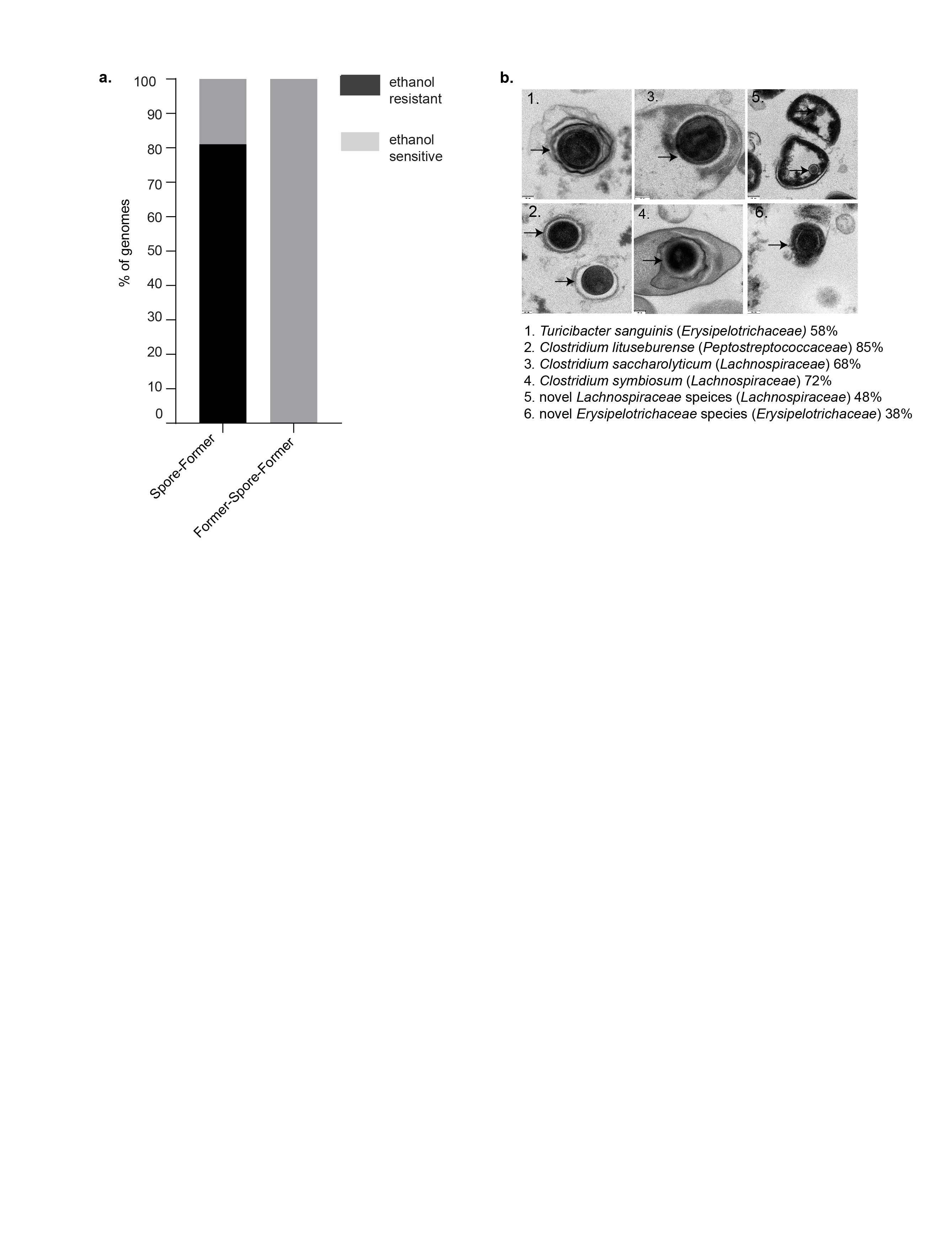


**Figure S3: Phenotypic validation of sporulation capability predictions**

Phenotypic validation of loss of sporulation using exposure of isolates to ethanol and isolation from ethanol treated faeces **(a)** and Transmission Electron Microscopy (TEM) **(b).** Spores were only identified in bacteria predicted to be spore-forming. Arrows indicates spores. Species name corresponding to TEM image is indicated along with corresponding family in brackets and sporulation signature score.

**
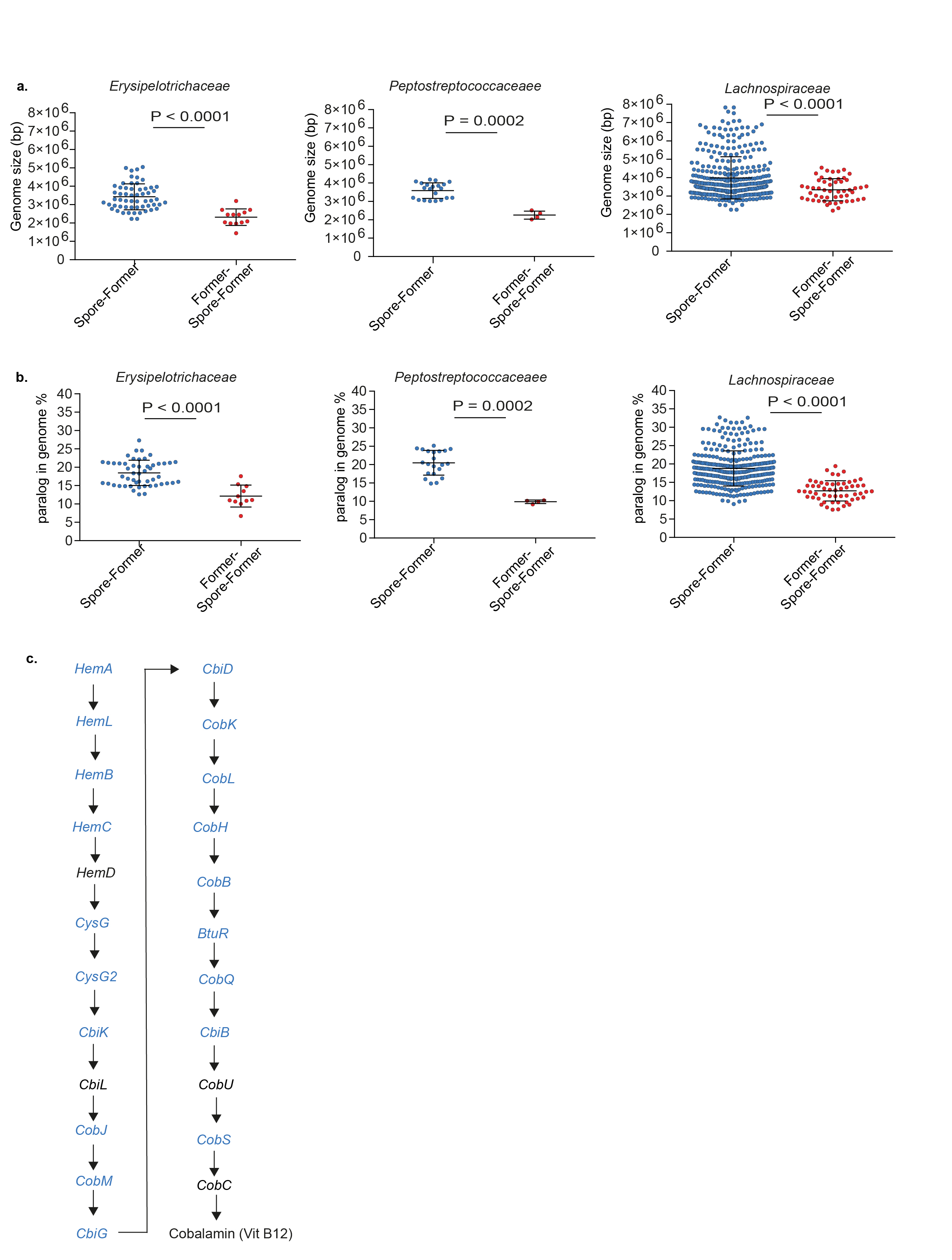
**

**Figure S4: Genome reduction and metabolic specialization during host-adaptation by gut Firmicutes**

Former-Spore-Formers (FSF) of host-associated bacterial families, *Erysipelotrichaceae, Peptostreptococcaceae* and *Lachnospiraceae* have more reduced genomes compared to Spore-Formers (SF) within the same family as measured by a smaller genome size **(a)** (*Erysipelotrichaceae* SF gut vs. FSF gut P< 0.0001, *Peptostreptococcaceae* SF gut vs. FSF oral P=0.0002, *Lachnopsiraceae* SF gut vs. FSF oral and rumen P< 0.0001, Mann-Whitney), mean with SD, and by encoding less paralogs **(b)** (*Erysipelotrichaceae* SF gut vs. FSF gut P<0.0001, *Peptostreptococcaceae* SF gut vs. FSF oral P=0.0002, *Lachnopsiraceae* SF gut vs. FSF oral and rumen P< 0.0001, Mann-Whitney) , mean with SD. **(c)** SF are enriched in 19 of 23 genes (marked in blue) directly required for cobalamin (Vitamin B12) biosynthesis unlike FSF which are not enriched for cobalamin biosynthesis genes.

**
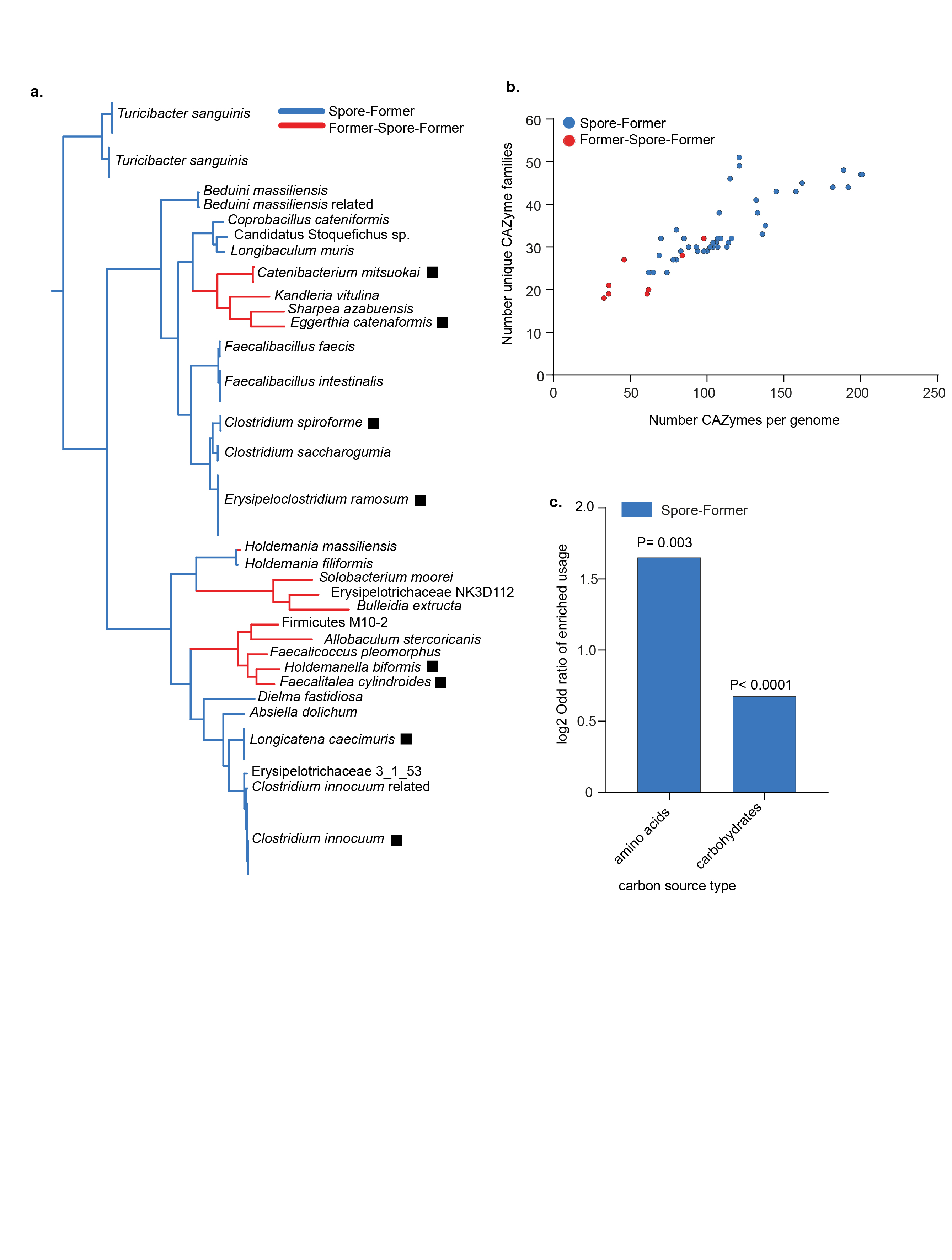
**

**Figure S5: *Erysipelotrichaceae* Former-Spore-Formers have a reduced carbohydrate metabolism profile compared to *Erysipelotrichaceae* Spore-Formers**

**(a**) Phylogeny of the *Erysipelotrichaceae* family, the 32 distinct species names are indicated. Species whose name is followed by a rectangle were selected for phenotypic analysis**.** Novel species are denoted by closest relative followed by term ‘related’. **(b)** *Erysipelotrichaceae* gut FSF encode a smaller number of total CAZymes and a smaller number of CAZyme families per genomes compared to *Erysipelotrichaceae* gut SF (P<0.0001 and P=0.0001 for total number and family number of CAZymes respectively, Welch’s t-test).  **(c)** The ability of FSF (n=4) and SF (n=4) to use 95 different carbon sources was tested. Carbon sources were grouped by category and the log2 odds ratio was calculated. Carbohydrates (45 in total) (P<0.0001) and amino acids (17 in total) (P=0.003, Fishers exact test) were utilised less by *Erysipelotrichaceae* FSF compared to SF. No carbon source group were utilised more by FSF compared to SF.

**
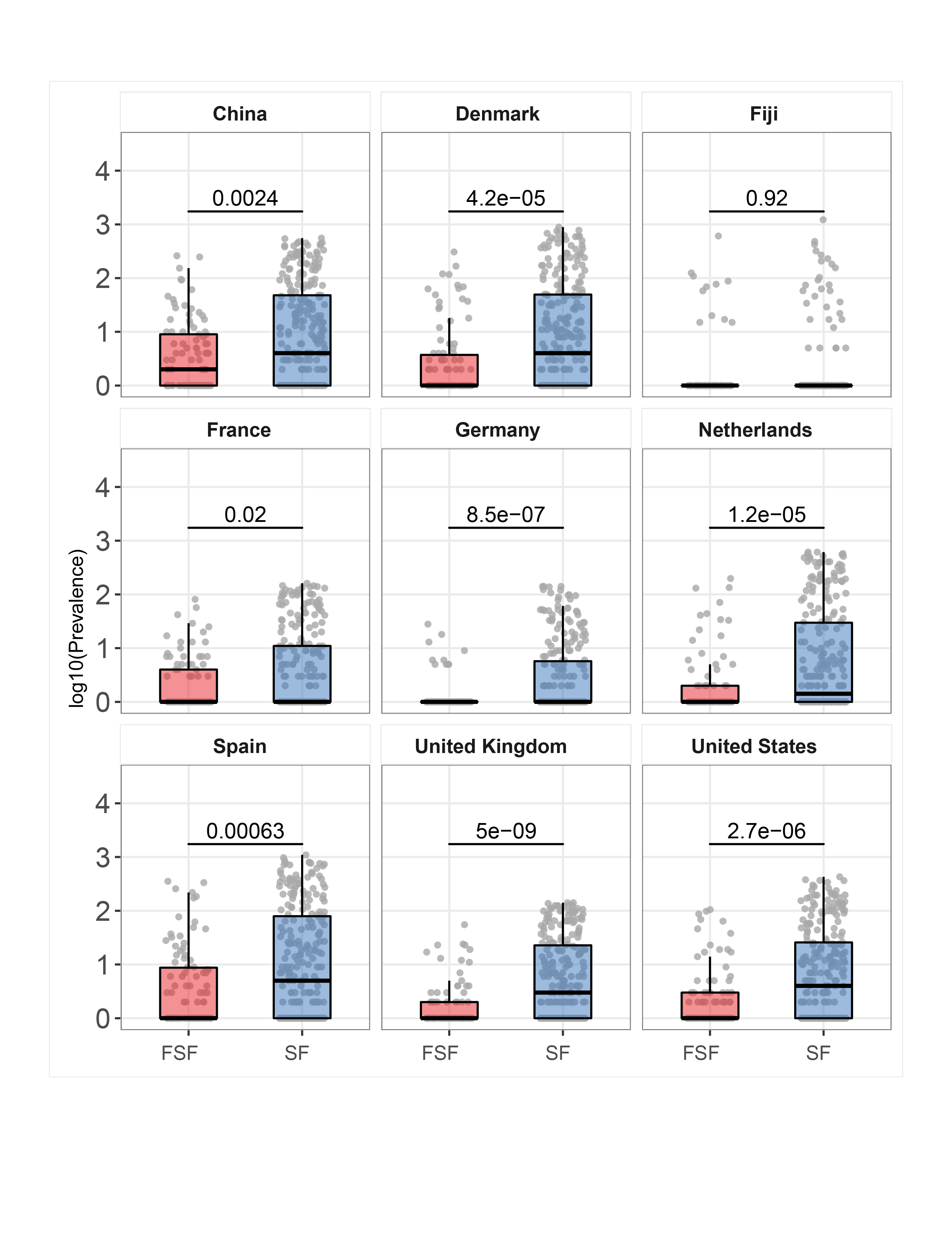
**

**Figure S6: Former-Spore-Formers are less prevalent than Spore-Formers in gut metagenomes from the same country**

Former-Spore-Formers (red) are less prevalent compared to Spore-Formers (blue) in gut metagenomes from the same country (P<0.05, two-tailed Wilcoxon rank-sum test for 8 of 9 countries tested). Only countries with more than 150 samples were included in the analysis. Each dot represents an individual species**.** Box lengths represent the IQR of the data, and the whiskers the lowest and highest values within 1.5 times the IQR from the first and third quartiles, respectively.


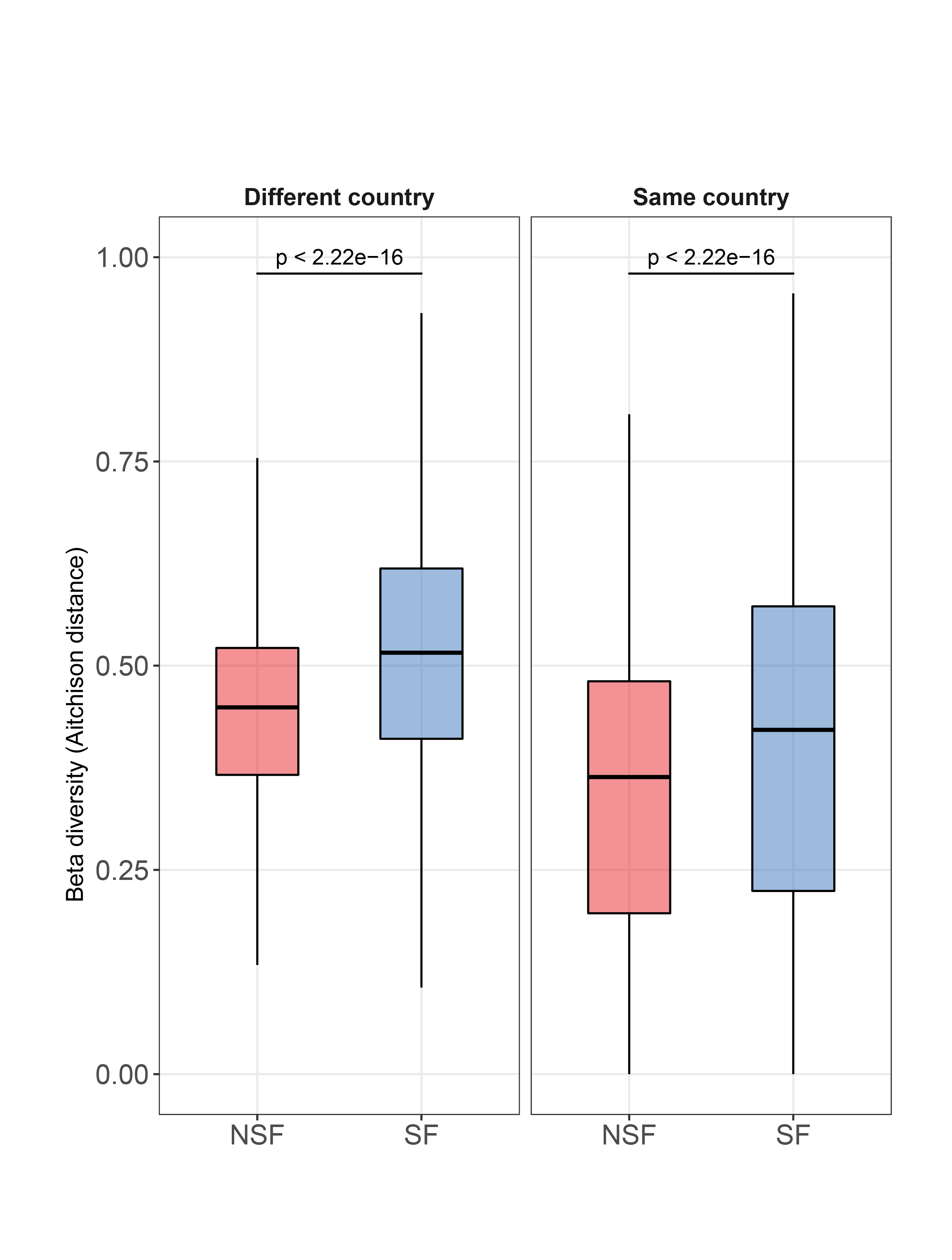


**Figure S7: Spore-formers contribute more to beta-diversity compared to non-spore-forming bacteria in the human intestinal microbiota.**

Beta-diversity (Aitchison distance) of metagenomes was calculated for spore-forming and non-spore-forming bacterial species both within the same country and between different countries. Spore-forming species contribute more to beta-diversity than non-spore-forming bacteria (two-tailed Wilcoxon rank-sum test).
